# Nitric Oxide: Its Generation and Interactions with Other Reactive Signaling Compounds

**DOI:** 10.3390/plants8020041

**Published:** 2019-02-12

**Authors:** John T. Hancock, Steven J. Neill

**Affiliations:** 1 Department of Applied Sciences, University of the West of England, Bristol BS16 1QY, UK; 2 Faculty of Health and Applied Sciences, University of the West of England, Bristol BS16 1QY, UK; steven.neill@uwe.ac.uk

**Keywords:** antioxidants, hydrogen gas, hydrogen peroxide, hydrogen sulfide, nitric oxide, reactive oxygen species

## Abstract

Nitric oxide (NO) is an immensely important signaling molecule in animals and plants. It is involved in plant reproduction, development, key physiological responses such as stomatal closure, and cell death. One of the controversies of NO metabolism in plants is the identification of enzymatic sources. Although there is little doubt that nitrate reductase (NR) is involved, the identification of a nitric oxide synthase (NOS)-like enzyme remains elusive, and it is becoming increasingly clear that such a protein does not exist in higher plants, even though homologues have been found in algae. Downstream from its production, NO can have several potential actions, but none of these will be in isolation from other reactive signaling molecules which have similar chemistry to NO. Therefore, NO metabolism will take place in an environment containing reactive oxygen species (ROS), hydrogen sulfide (H_2_S), glutathione, other antioxidants and within a reducing redox state. Direct reactions with NO are likely to produce new signaling molecules such as peroxynitrite and nitrosothiols, and it is probable that chemical competitions will exist which will determine the ultimate end result of signaling responses. How NO is generated in plants cells and how NO fits into this complex cellular environment needs to be understood.

## 1. Introduction

Since nitric oxide (NO) was mooted to be an important signaling molecule in animals in 1987 [1], and with the subsequent reporting of its role in plant signaling [2,3,4], there has been extensive work on investigating its function in plants.

Higher plants would have evolved through a lineage that would have been exposed to a range to toxic and reactive compounds and have therefore adapted to encompass them into their normal metabolism [5]. NO, along with reactive oxygen species (ROS) such as the superoxide anion (O_2_^·-^) and hydrogen peroxide (H_2_O_2_), along with hydrogen sulfide (H_2_S), works as part of a suite of relatively reactive small molecules in cells which help to control the cell’s activity and the function of proteins. NO has been implicated in seed germination [6], root development [7], stomatal closure [8], pathogen challenge [9], plant reproduction [10,11] and stress responses [12]. Therefore, how NO is produced, perceived and leads to a range of effects is important to unravel.

The generation of NO in plants remains controversial, as discussed below, while the measurement of NO [13] in plant materials is still contentious, and often it is not possible to give its sub-cellular location or quantification. This can itself lead to problems with interpretation, as it is not known if NO accumulates to significant, perhaps what could be referred to as threshold, levels, or whether the accumulation of NO is compartmentalized, as reported for other signaling molecules [14], such as cAMP [15,16] and Ca^2+^ [17] but also including ROS and redox signaling [18,19]. Therefore, the idea of compartmentalisation is important to consider here. It is often difficult, therefore, to interpret the data generated. On top of this, NO will react with other signaling molecules, and this makes it difficult to understand fully how NO integrates into a complex signaling pathway. This is also further discussed below.

## 2. Nitric Oxide Generation in Plant Cells

There seems to be little doubt that plant cells generate NO and are able to respond to it. Therefore, multiple routes to NO accumulation have been suggested, including some that are enzyme-dependent and others that are enzyme-independent [20,21].

One of the major sources of NO is the enzyme nitrate reductase (NR) [22,23]. It has been shown to be important, for example, in the control of stomatal closure [24]. *Arabidopsis thaliana* has two isoforms of NR, and it is thought that both are important in signaling [25,26]. Furthermore, other proteins may interact to create nitrite-dependent enzymes as well [22].

Another enzyme which can generate NO, albeit under hypoxic conditions, is xanthine oxidoreductase (XOR) [27], while other molybdenum-based enzymes may also be important [22]. However, the enzyme which has attracted most attention, perhaps not surprisingly, is nitric oxide synthase (NOS). Despite early reports of the isolation of a NOS from higher plants, it became apparent that the protein which directly produces NO was not identified [28]. To date, this remains controversial.

In lower plants, NOS homologues have been identified [29]. Two green algae genomes showed evidence of sequences for NOS, *Ostreococcus tauri* and *Ostreococcus lucimarinus* [30]. The *O. tauri* sequence was 45% similar to human NOS and the structure was most similar to eNOS. On characterising this enzyme, it was found that the k_m_ for L arginine, the likely substrate for this NOS enzyme, was found to be 12 ± 5 µM, suggesting that it might have physiological relevance [30]. Such data give hope for finding such an enzyme in higher plants. However, the literature on the nature of a plant NOS has been reviewed widely, and it has been argued previously that higher plants do not contain a NOS enzyme [31,32]. More convincing is the genomic search that was reported [33]. Here, the search involved data sets from the 1000 Plants (1KP) international consortium. No typical NOS sequences were found when 1087 sequenced transcriptomes from land plants were investigated. In contrast to this, 15 of the 265 algal species analyzed showed evidence of NOS sequences. The authors concluded that land plants must produce NO using a different mechanism to that found in animals [33]. This makes it hard to explain much of the data that has been published on NOS-like enzymes in plants, such as a recent study on barley root tips [34] where the NOS inhibitor N(ω)-nitro-L-arginine methyl ester (L-NAME) was shown to have effects. Such work leads researchers to refer to a NOS-like enzyme in plants, but as no homologue, at the gene or protein level, to a mammalian NOS has been reported in any higher plant, it is suggested here that the term NOS-like should not be used and such enzymes and proteins should be referred to as nitric oxide generating (NOGs).

If an enzyme were to generate NO in manner similar to that reported for mammalian NOS, there should be identifiable aspects. Butt et al. [35] used a proteomic approach to identify plant proteins which cross-reacted with mammalian NOS antibodies. Using 2-D gels of extracts from *Zea mays* L. they reported that 20 proteins were immunoreactive following Western blot analysis. Fifteen of the proteins were identified using matrix-assisted laser desorption/ionization time-of-flight mass spectrometry and found not to be related to NO metabolism. Although five proteins remained unidentified, the authors concluded that the immunological techniques so far used were not sufficient to infer the presence of a plant NOS protein [35]. For an up-to-date summary of the discussion of the presence of NOS in plants, see Santolini et al. [32] or Astier et al. [21].

**Figure 1:**
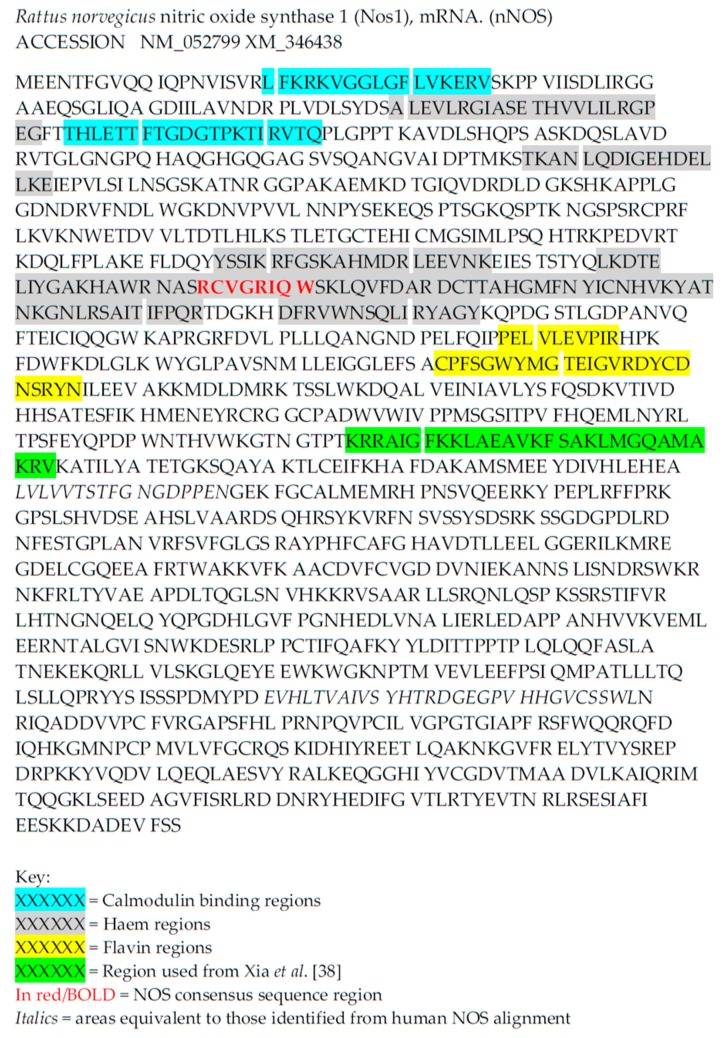
Areas of the rat NOS sequence used to search for higher plant NOS-like proteins. Findings shown in Table 1.

However, it cannot be assumed that all elements of a mammalian NOS should be identifiable. OtNOS lacks the autoregulatory control element, suggesting that it is most closely related to the iNOS isoform in mammals [30]. This may also suggest that looking for a similar domain in a higher plant NOS is futile. Here, as an example of the sorts of aspects that could be looked for, a bioinformatics approach was used. This way of searching for a NOS-like enzyme is predicated on the fact that there should be domains or motifs which are important for NOS-like function, and therefore there should be some level of conservation in these sequences, albeit perhaps hard to find. The mammalian enzymes contain an oxidase domain, a reductase domain and regions which are able to interact with calmodulin [36]. Here, small stretches of sequence have been used to search for possible NOS-like candidates in Arabidopsis and Oryza (Table 1). These relatively short sequences have been derived from the work of others [37,38], such as those looking for NOS-interactions in rat NOS, as well as using the Prosite [39] NOS signature and sequences identified from alignments of the three human NOS proteins using ClustalOmega [40] (data not shown). The relative positions of such sequences within the rat nNOS peptide sequence is shown in Figure 1.

If a plant NOS-like enzyme were to function in a manner similar to a mammalian one, it should have a reductase domain capable of oxidizing NAPDH and having a flavin prosthetic group, such as flavin mononucleotide (FMN). The mammalian NOS reductase is homologous to that of the P450 family of enzymes [41]. Plants have reductases which are similar. Arabidopsis has two proteins which can identified as p450 reductases: NP_001190823 and NP_194750. Some of the short sequences used in Table 1, such as eNOS 952–980, found evidence of reductases in the plant genomes searched. It is therefore possible that any NOS-like enzyme in plants does not have a dedicated reductase, but can draw electrons from other reductases, which are possibly multifunctional.

If there is no need for a dedicated reductase domain, this is almost certainly not true for the oxidase domain. To generate NO, this is the active site that would need to exist. It is very possible that the plant NOS-like protein may only be an oxidase domain, lacking a reductase. It has been reported that bacterial NOS enzymes are indeed like this, lacking a reductase but using electron donation from a nonspecific reductase [42]. Therefore, a search for the oxidase domain is important.

Using the NOS signature from the rat nNOS sequence (NM_052799 XM_346438) in Blastp at NCBI had the highest score hit of hypothetical protein OsI_24933 [*Oryza sativa* Indica Group] ID: EAZ02807.1. This had the following match:


        Query 1   RCVGRIQW 8
        RC G IQW
        Sbjct 224  RCTGKIQW 231
      

The same match was found for an *Arabidopsis thaliana* hypothetical protein (amino acids 115–122: Table 1). Others on the Blastp output are annotated as F-box kelch-repeat proteins. Putting both the *Oryza* and *Arabidopsis* sequences through Prosite revealed nothing of significance; only phosphorylation sites and other Prorules for post-translational modifications. Therefore, these hypothetical proteins look unlikely to be able to act as part of a NOS protein.

Using the NOS signature from the Prosite ProRule data (PS60001) revealed nothing in plants of significance, but it did pull out NOS-like sequences from a range of other organisms, including Staphylococcus and insects. Therefore, there is little evidence of this short NOS signature sequence being in either Arabidopsis or Oryza databases, at least to date.

NOS is likely to interact with other peptides, and this would be a way to identify important functional regions. The calmodulin-interacting regions (CaM) from the rat sequence revealed nothing of note. When the three human NOS sequences were aligned, these CaM motifs were not represented in all NOS peptides, and therefore it could be argued that they are not essential, and not finding them does not rule out the presence of a plant NOS. Others have looked for other interacting regions as well [37,38]; for example, between the FMN and oxidase domains. Taking interesting sequences such as those thought to be involved in protein interactions from the literature also failed to reveal a likely NOS sequence in Arabidopsis or Oryza (Table 1).

It can be concluded so far, in that case, that there is no significant evidence from the sequence searching of a NOS-like protein in two plant sequences for which major genome sequencing projects have been undertaken [43,44].

NOS in other species is not a stand-alone protein, but has interacting partners. NOS-interacting proteins can be found in the literature (Table 1), such as nostrin [45], carboxyl terminal PDZ ligand [46] and NOS-interacting protein [47]. Searching for evidence of such proteins in plant genomes may give circumstantial evidence of a NOS-like protein in plants. The nostrin sequence found plant proteins which can interact, perhaps through SH3 domains (Table 1). However, the most intriguing fact was that the *Homo sapiens* NOS interacting protein isoform (NP_057037) was revealed in both the Arabidopsis and Oryza data proteins, which have already been annotated as NOS-interacting proteins (XP_020890108.1 & XP_006649867.1, respectively). Such proteins may be used as lures to find interacting partners in plant extracts, some of which may have NOS-like activity.

Overall, the bioinformatic searching carried out here, although by no means exhaustive, showed no clear evidence of a NOS-like protein in plants, although elements such as a reductase do clearly exist. These data are not contrary to those found and reported by others [33].

## 3. Interactions of Nitric Oxide with Other Reactive Signals

When the chemistry of nitric oxide is discussed, it is often assumed that this involves the radical form: NO^·^. However, with the loss or gain of an electron, other forms are nitroxyl (NO^−^) and nitrosonium (NO^+^) ions [48]. It is important to appreciate that NO will not be generated in cells in isolation. It is often produced in response to a stress, and as such, other signals will be accumulating at the same time, including ROS and H_2_S. If cadmium ion stress in plants is taken as an example, the cellular response includes the generation of NO and ROS [49], as well as H_2_S [50], all presumably being accumulated in the same sub-cellular location, such as the cytoplasm. Therefore, it is important to consider how NO may interact with other compounds that are present.

**Figure 2:**
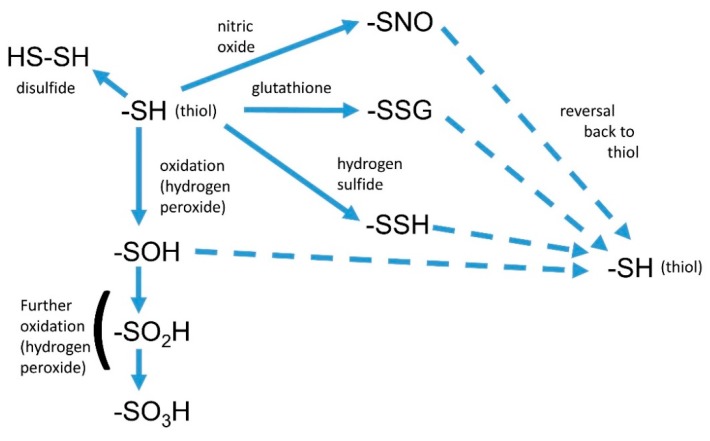
Some post-translational modifications of thiol groups. These include S-nitrosation and oxidation. Many modifications are reversible, and so are akin to phosphorylation.

One of the main downstream effects of NO is the post-translational modifications of thiols (Figure 2) and other amino acids such as tyrosine. *S*-nitrosation (often referred to as *S*-nitrosylation) is the modification of the –SH group to –SNO [51], which may cause a conformational change in the protein, with a concomitant change in activity or function. However, the thiol group may also be modified by oxidation, *S*-persulfidation by H_2_S, glutathionylation by GSH, or reaction with another thiol to create a disulfide (reviewed previously [52]), and so a reaction with NO is not necessarily the outcome. With such a range of possible reactions, the actual resultant change seen will be dependent on the local concentrations of reactants and the kinetics of the possible reactions.

The protein modification by NO is, however, an important signaling process. Many proteins in plants have been identified as being nitrosated [51], with a good example being glyceraldehyde 3-phosphate dehydrogenase (GAPDH). In mammalian cells, it has been shown that on *S*-nitrosation, the enzyme translocates to the nucleus, thus abandoning its role in glycolysis to take up a new role in the control of gene expression [53]. In plants, GAPDH has also been shown to be *S*-nitrosated, and cytosolic GAPDH can interact with nuclear DNA, specifically to a partial gene sequence of NADP-dependent malate dehydrogenase [54]. However, GAPDH can also be modified through oxidation by H_2_O_2_ [55] and in addition be *S*-persulfidated by H_2_S [56], with the latter known to lead to its translocation to the nucleus. Clearly, there is competition between reactive signals in cells [52], and it cannot be assumed that NO signaling will dominate. However, methods to identify thiol modifications will help to unravel such signaling [57,58].

*S*-nitrosation also has a role in mediating the interplay between NO and other reactive signaling mechanisms, such as those involving ROS. For example, key enzymes which generate ROS, such as NAPDH oxidase, can be modified by NO. It has been reported that RBOHD is *S*-nitrosated at Cys890 which inactivates the enzyme and thus reduces its ROS-generating activity [59]. Therefore, NO has an important role in controlling ROS levels and hence the potential downstream signaling here.

The second important modification of proteins brought about by NO is tyrosine nitration [60], and again this may lead to alterations of function. As with *S*-nitrosation, NO interaction will lead to conformational changes in the protein and commensurate changes in activity, either increased or decreased. Some of these modifications may have the result of altering other signaling pathways mediated by other reactive signals; for example, tyrosine nitration can alter superoxide dismutase (SOD) activity and hence ROS signaling [61].

**Figure 3:**
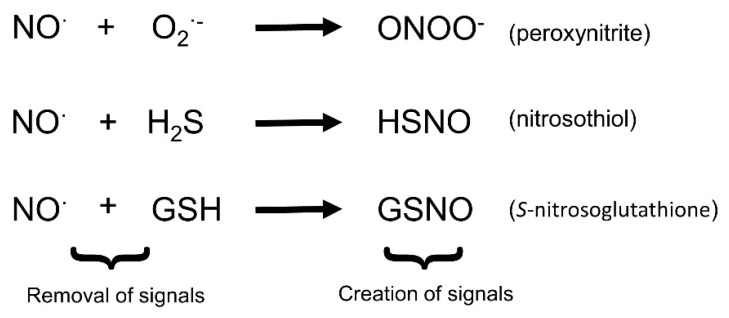
Some of the reactions in which NO can be involved, often leading to new signaling molecules.

As NO and ROS are produced in the cell at the same time, it is important to consider their interaction and the ramifications of this chemistry. The most well-known reaction of NO and ROS it that between the superoxide anion and NO which produces peroxynitrite (ONOO^−^) (Figure 3). This has two potentially important outcomes. Firstly, the reaction removes both O_2_^·-^ and NO from the cell or the cell’s environment, thereby reducing the bio-availability of both. Thus both ROS-dependent signaling, perhaps through H_2_O_2_, and NO signaling would be reduced. Secondly, there is a new compound produced which itself can act as a signaling molecule [62], perhaps giving a different response than would have resulted from ROS or NO signaling.

The generation of NO will also be into an environment rich in antioxidants. NO may affect the activity of enzymatic antioxidants, as mentioned above, where NO, through a peroxynitrite-mediated mechanism, altered SOD activity [61], and hence reduced the cell’s capacity to remove superoxide anions and produce H_2_O_2_, with the latter being important in signaling. In a similar manner, NO can alter catalase activity [63], thus lowering the cell’s capacity to remove H_2_O_2_, perhaps prolonging ROS-mediated signaling.

A large part of the antioxidant capacity of the cell is due to the presence of low molecular-weight antioxidants. There are a range of small low molecular-weight thiols in cells [64], but one of the most important is glutathione [65]. This exists in the reduced state (GSH) and the oxidized state (GSSG), with the ratio of these compounds, along with the total GSH+GSSG concentration, being partly responsible for the maintenance of the intracellular environment in a very reduced state [65], probably below −200 mV. It is possible that the presence of NO—as it is a redox compound—will lead to the intracellular redox status being altered. It is known that the intracellular redox environment is not static and becomes more oxidizing if cells are in an apoptotic state [65], but it is also possible that the redox environment determines the state of any NO couple and hence the longevity of any NO species, as previously discussed [64,66]. In some cases, for example as the cell becomes more oxidizing, the presence of NO^·^ will be prolonged, and so this will enhance NO^·^-mediated signaling.

Importantly, NO and glutathione can react together to produce GSNO. This potentially has the capacity to reduce GSH/GSSG levels in cells, and hence potentially alter the intracellular redox environment, especially if the reaction is compartmentalized. The reaction will also remove NO from directly partaking in further signaling. However, GSNO has important roles as well. GSNO can act as a donor and therefore a reservoir of NO, and it has been suggested that GSNO can mediate some NO effects [67], having distinct and overlapping molecular targets when compared to NO itself. GSNO has also been mooted as an important mechanism to transport NO around organisms [68], perhaps through the vasculature system of plants. To terminate GSNO-mediated signaling, it can be removed by the action of GSNO reductase [69,70], which would lower the bioavailability of NO.

Another reactive signal which may interfere with NO signaling is hydrogen sulfide (H_2_S). H_2_S has recently been found to be an important signaling molecule in both animals and plants [71,72,73]. It is produced in response to a range of stresses, such as cadmium ions, as mentioned above [50]. H_2_S can react directly with NO to produce nitrosothiol (Figure 3). As discussed above, this will reduce the bioavailability of both H_2_S and NO, but it will also create a new molecule with potential signaling effects [74]. H_2_S will also increase GSH levels in cells [75] and therefore may have the potential to alter the accumulation of GSNO.

Lastly, it has recently been suggested that signaling in animals and plants may involve hydrogen gas (H_2_) [76]. The presence of H_2_ may alter antioxidant levels in cells [77] and so indirectly alter NO metabolism. However, there is also potential for a more direct interaction with H_2_ and some nitrogen compounds [78,79]. Certainly, H_2_ has been shown to have effects in plants [80,81] and has touted as a future treatment for plants [82]. NO has been reported to be needed for some of the H_2_ gas effects [83,84] and no doubt more interactions between NO and H_2_ signaling will be revealed in the future.

## 4. Conclusions and the Future

NO is a key signaling molecule in plants, being important in plant reproduction [85], development [86] and plant cell death [87]. However, the production of NO in plants remains controversial. Enzymes such as NR are known to be important [23], while others such as XOR may be involved. Much data points to the existence of a NOS-like enzyme being present in higher plants, and although there is such an enzyme in algae [29], the search for homologues in higher plants remains elusive [33], and it appears that such an enzyme really does not exist. It is possible that a novel peptide has oxidase-type activity which can produce NO, receiving electrons from a less specific reductase, as seen in bacteria [42], but if such a peptide does exist, it is very difficult to identify.

If there is an oxidase-type enzyme in higher plants it would need to obtain its electrons from somewhere; most likely a reductase, as seen with P450. As can be seen in Table 1, such reductases in plants do exist and might serve this function. Furthermore, putative NOS-interacting proteins have been identified in plants, as listed in Table 1. Therefore, by concentrating on proteins which are most likely to interact with an oxidase-like protein and using these as bait in purification experiments, it is possible that the future may see a novel NO-generating oxidase being discovered in higher plants. However, with divergent evolution of plants and animals, and the fact that plants appear to have other NO generating pathways such as nitrate reductase, it may be that such an NO-producing oxidase does not exist.

The role of NO is also complex and not fully understood. NO is made in plants cells in response to the same cues that initiate the generation of ROS and H_2_S, and so NO will not work in isolation. The reaction of NO with ROS or H_2_S will lower the bioavailability of NO, but also produce new signaling molecules, such as peroxynitrite [62] and nitrosothiols [74], which will have their own outcomes. The impact of NO on the cellular redox poise, especially if compartmentalized, needs to be considered, as does the impact of the redox environment on the NO metabolism that may ensue [64]. NO will interact with antioxidants, such as glutathione, which may even facilitate its organismic transport [68]. Furthermore, one of the main actions of NO is to chemically modify proteins, for example through *S*-nitrosation, but this may not be possible if other reactive compounds such as H_2_S or ROS have already modified the relevant thiol. Therefore, the downstream actions of NO cannot always be assumed.

In conclusion, two major barriers exist to the progression of NO research in plants. Firstly, the controversy surrounding the presence of NO-generating enzymes needs to be resolved. Here, is it suggested that the term NOS-like is dropped to avoid continual confusion by drawing parallels with the mammalian system, as clearly the homology does not exist. The term nitric oxide-generating (NOG) would be more accurate. Secondly, the way NO is interwoven into the signaling of other important reactive chemicals needs to be understood. Is NO metabolism compartmentalized in such a way that ROS, GSH or H_2_S do not interfere, or is there a competition between all these signals, keeping each other in check, as already been mooted [88]? Until such issues are resolved, the true nature of the role of NO in plants will remain elusive.

## Figures and Tables

**Table 1:** Sequences used to search for matches in *Arabidopsis* and *Oryza* using Blastp and tBlastn. Areas used from the rat nitric oxide synthase (nNOS) sequence are highlighted in Figure 1.

Source of Sequence	Sequence Name	Sequence	Reference/ Source for Sequence	Significant Find/Comment
NCBI	NOS signature	[GR]-C-[IV]-G-R-[ILS]-x-W	prosite.expasy.org/ PS60001	No significant sequence identified using ScanProsite/ previously found in a range of species including Staphylococcus, insects and mammals.
Rat nNOS	NOS signature	-RCVGRIQW-	[37]	Hypothetical protein OsI_24933 [*Oryza sativa* Indica Group] ID: EAZ02807.1, Length: 515, Identity 75%, Query cover 100%. Hypothetical protein AXX17_AT1G30490 [Arabidopsis thaliana] ID: OAP19388.1, Length: 398, Identity, 75%, Query cover 100%.
Rat nNOS	FMN subdomain 538–547	-PELVLEVPIR-	[37]	No significant sequence identified
Rat nNOS	FMN subdomain 582–605	-CPFSGWYMGTEIGVRDYCDNSRYN-	[37]	No significant sequence identified
Rat nNOS	Haem domain 80–102	-ALEVLRGIASETHVVLILRGPEG-	[37]	No significant sequence identified
Rat nNOS	Haem domain 187–203	-TKANLQDIGEHDELLKE-	[37]	No significant sequence identified
Rat nNOS	Haem domain 366–386	-YSSIKRFGSKAHMDRLEEVNK-	[37]	No significant sequence identified
Rat nNOS	Haem domain 396–465	-LKDTELIYGAKHAWRNASRCVGRIQW SKLQVFDARDCTTAHGMFNYICNHVKY ATNKGNLRSAITIFPQR-	[37]	No significant sequence identified /NOS consensus sequence underlined, but not found in plants.
Rat nNOS	Haem domain 471–485	-DFRVWNSQLIRYAGY-	[37]	No significant sequence identified
Rat nNOS	CaM domain 20–36	-LFKRKVGGLGFLVKERV-	[37]	No significant sequence identified
Rat nNOS	CaM domain 105–124	-THLETTFTGDGTPKTIRVTQ-	[37]	No significant sequence identified
Human iNOS	509–537	-KRREIPLKVLVKAVLFACMLMRKTMASRV-	[38]	Poor homology in some *Oryza* sequences/R536 important in human (underlined)/ -SRV- present
Rat nNOS	725–753	-KRRAIGFKKLAEAVKFSAKLMGQAMAKRV-	[38]	Poor homology in some *Oryza* sequences /R752 important (underlined) in rat
Mouse iNOS/FMN domain	532–694	-VRATV…PKRFT-	Derived from [37]	Os08g0243500, partial [*Oryza sativa* Japonica Group] ID: BAF23260.1, Length: 651, Identity 32%, Query cover 82%. NADPH--cytochrome P450 reductase [*Oryza sativa* Japonica Group] ID: XP_015650780.1, Length: 719, Identity 32%, Query cover 82%. PREDICTED: NADPH-dependent diflavin oxidoreductase 1 [*Oryza brachyantha*] ID: XP_006659755.1, Length: 625, Identity 35%, Query cover 89%. Hypothetical protein OsJ_30318 [*Oryza sativa* Japonica Group] ID: EEE70211.1, Length: 795, Identity 32%, Query cover 89%. Others similar can be identified.
eNOS (human)	566–585	-LVLVVTSTFGNGPPENGES-	Derived from human Clustal Omega e/i/n NOS	No significant sequence identified
eNOS (human)	952–980	-EIHKTVAVLAYRTGDGLGPLHYGVCSTWL-	Derived from human Clustal Omega e/i/n NOS	Evidence of being part of a oxidoeductase or P450 reductase in plants: for example XP_015696451.1 & XP_006653834.1(both have 45% identical over 96% coverage) from Oryza: CAA46814.1 & NP_194183.1 (both 67% identical over 41% coverage) from Arabidopsisl.
*Nitric Oxide Synthase Related Proteins/Peptides*				
Nostrin isoform 2 [*Homo sapiens*]: NP_001034813	Full sequence	MRDPLT…NTATKA	https://www.ncbi.nlm.nih.gov/protein/NP_001034813.2/ & [45]	SH3 domain-containing protein 3 [*Arabidopsis lyrata* subsp. lyrata] ID: XP_002868013.1, Length: 351, Identity 37%, Query cover 15% Os04g0539800 [*Oryza sativa* Japonica Group] ID: BAF15352.2, Length: 115, Identity 48%, Query cover 10% Putative protein [*Arabidopsis thaliana*] ID: CAB53647.1, Length: 330, Identity 26%, query cover 30%.
Carboxyl-terminal PDZ ligand of neuronal nitric oxide synthase protein isoform 1 [*Homo sapiens*]. NP_055512	Full sequence	MPSKT…DDEIAV	https://www.ncbi.nlm.nih.gov/protein/NP_055512 & [46]	No significant sequences identified/PH-like superfamily predicted.
Nitric oxide synthase-interacting protein isoform 1 [*Homo sapiens*]. NP_057037	Full sequence	MTRHG…SRPVMGA	https://www.ncbi.nlm.nih.gov/protein/NP_057037 & [47]	PREDICTED: nitric oxide synthase-interacting protein [*Oryza brachyantha*] ID: XP_006649867.1, Length: 305, Identity 32%, Query cover 96% E3 ubiquitin-protein ligase CSU1 [*Oryza sativa* Japonica Group] ID: XP_015630570.1, Length: 305, Identity 32%, Query cover 96%. Phosphoinositide binding protein [*Arabidopsis thaliana*] ID: NP_564781.1, Length: 310, Identity 31%, Query cover 98%. Nitric oxide synthase-interacting protein homolog [*Arabidopsis lyrata* subsp. lyrata] ID: XP_020890108.1, Length: 310, Identity 31%, Query cover 98%.
